# Bone mineral loss and cognitive impairment: The PRESENT project

**DOI:** 10.1097/MD.0000000000012755

**Published:** 2018-10-12

**Authors:** Hyun Goo Kang, Hyun Young Park, Han Uk Ryu, Seung-Han Suk

**Affiliations:** a Department of Neurology, Chonbuk National University Hospital, Jeonju; b Department of Neurology, Wonkwang University School of Medicine, Institute of Wonkwang Medical Science, Iksan; c Department of Neurology, Wonkwang University Ansan Municipal Geriatric Hospital and Center for Prevention of Stroke and Dementia, Ansan, South Korea.

**Keywords:** bone mineral density, cognitive impairment, dementia, osteoporosis, risk factor

## Abstract

Low bone mineral density (BMD) is correlated with Alzheimer's disease and its severity, but the association remains unclear in adults (≥50 years) without a history of stroke or dementia.

We assessed BMD and cognitive function using the Mini-Mental Status Examination (MMSE) in 650 stroke- and dementia-free subjects (≥50 years) who were recruited for an early health check-up program between January 2009 and December 2010.

The mean age was 62.9 ± 8.0 years and mean MMSE score was 27.6 ± 3.6. A total of 361 subjects had reduced BMD: 197 (30.3%) had osteopenia and 154 (23.6%) had osteoporosis, based on criteria of world health organization. A total of 5.4% of the male subjects had osteoporosis, versus 19.8% of the female subjects. After adjusting for age, sex, education, and other possible confounding factors such as hypertension, diabetes mellitus, and smoking, the estimated odds ratio for cognitive impairment was 1.72 for the osteopenia group (95% confidence interval [CI] 1.09–2.14, *P* = .019) and 2.81 for the osteoporosis group (95% CI 1.78–4.45, *P* < .001).

Low BMD is correlated with cognitive impairment in community-dwelling adults aged 50 years and above without any medical history of stroke or dementia, especially in women. A community-based, early life, preventive osteoporosis education campaign might decrease the incidence of dementia.

## 1 Introduction

Recently, dementia has been detected in 14% to 20% of the elderly. Approximately 4.6 million people are diagnosed with dementia each year, with a 2-fold increase in the disease prevalence every 20 years.^[1]^ With aging and physical inactivity, there is a reduction in muscle mass, muscle strength, and bone mineral density (BMD). Over 200 million people suffer from osteoporosis across the world. Low BMD is correlated with Alzheimer's disease (AD) and its severity,^[1–6]^ and several studies have shown a relationship between low BMD and cognition in elderly women having common risk factors such as old age, sex, vitamin D3 deficiency and low estrogen.^[3,4,7]^ Low BMD and cognitive impairment often occur together, and osteoporosis is associated with progression of cognitive impairment.

However, to date, the correlation between BMD and cognitive function in community-dwelling healthy adults aged 50 years and over remains unclear, especially in men. In this study, we investigated the correlation between abnormal BMD and cognitive impairment in subjects without dementia or stroke, in order to generate basic data on the risk factors for cognitive impairment. We collected data from the Prevention of Stroke and Dementia (PRESENT) project, in a community setting. The PRESENT project is an ongoing regional government project that was initiated in July 2007 for the PRESENT through public education, public relations, early medical check-ups, and research, in Ansan City, Gyeonggi Province, Korea.

The aim of the present study was to evaluate the association of BMD and cognitive impairment over a 3-year follow-up period among community-dwelling adults in Korea. This report may be a valuable resource for establishing healthcare policies and prevention strategies for dementia among healthy adults.

## 2 Materials and methods

### 2.1 Study population

As part of the PRESENT project, subjects who were healthy and over 50 years old but not diagnosed with stroke or dementia were included. The study area was Ansan city Gyeonggi-do, South Korea, and sampling was performed by systematic random selection, after receiving a list of residents over 50 years old in Ansan City from the city government. As described previously,^[8]^ data collection was conducted from January 2009 to December 2010 and involved 2 steps. First, systematic random sampling with administrational support from the regional government was performed in 2009. Among the baseline cohort (n = 119,359) aged between 50 and 75 years, we contacted every 100th person using a registered list received from a regional government office (the PRESENT project is a regional government-run project). Telephone interviews were conducted by trained research nurses. If a potential participant could not be contacted, declined to participate, had moved, or had a history of stroke/dementia, we moved on to the next person on the list. Out of 1240 initial candidates, 525 could not be contacted, 366 declined to participate, 26 had moved, and 23 had a history of stroke or dementia. As a result, 300 participants were selected in the first step. In the second step, 350 subjects without any history of stroke or dementia were selected from volunteers of a low socio-economic background or who earned <200% of the minimum cost of living and wanted a health check-up in 2010 (Table 1). All patients provided informed consent for processing their anonymized data according to a protocol approved by the Institutional Review Board of Wonkwang University Hospital (WKIRB-201611-BM-071).

**Table 1:**
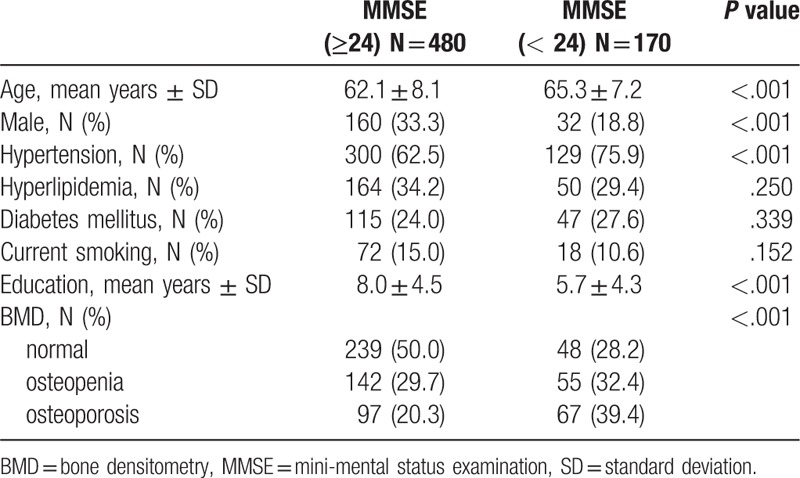
Demographic characteristics of subjects.

### 2.2 Baseline assessment

All participants received neurological examinations and in-person evaluations by a neurologist and trained research nurses. The in-person evaluation included medical questionnaires with questions regarding history of smoking, alcohol consumption, hypertension, diabetes, hyperlipidemia, stroke, and dementia. Questions concerning the education levels of participants were also included. Cognitive functions were measured in all the participants using the mini-mental status examination (MMSE), a widely used screening test, administered by psychologists and trained research nurses. Cognitive impairment was defined as an MMSE score of less than 24.^[9]^

### 2.3 BMD

All 650 participants were assessed for BMD using dual-energy X-ray absorptiometry. The GE LUNAR DPX-Bravo (GE health care, Buckinghamshire, UK) was used for the lumbar spine and femur. We divided BMD categories into 3 groups by T-score: normal, individuals with osteopenia, and individuals with osteoporosis. The T-score is the number of standard deviations (SD) below the average for a young adult at peak bone density. A T-score between +1 and −1.0 is considered normal or healthy, between −1.0 and −2.5 indicates osteopenia, and −2.5 or lower indicates osteoporosis according to the World Health Organization Task Force for Osteoporosis.^[8]^ A diagnosis of low BMD was based either on low T-scores of 2 parts at the same time, or a low T-score in any part of the lumbar spine or femur.

### 2.4 Statistical analysis

The subjects were classified into groups with more than 24 points or less than 24 points on the MMSE. Demographic, clinical, and BMD values were compared between the 2 groups. Clinical variables were analyzed using independent *t* tests, χ^2^ tests, and 1-way analysis of variance (ANOVA). Logistic regression analysis was conducted to determine independent factors of cognitive impairment after adjustment for potential confounders, including sex, age, hypertension, diabetes, current smoking status, education, and BMD. To avoid variable selection caused by multicollinearity correlations, only variables that showed *P* < .1 in univariate analysis were included in the multivariate logistic regression model. A 2-sided *P* value of <.05 was considered statistically significant. All statistical analyses were performed using SPSS 13 (IBM Corp., Armonk, NY).

## 3 Results

Six hundred and fifty adults aged 50 years and above (458 females and 192 males) completed all evaluation processes. We divided the subjects into 2 groups based on their MMSE scores. The demographic characteristics of subjects are shown in Table 1. Patient's age (65.3 ± 7.2 versus 62.1 ± 8.1 years, *P* < .001), the prevalence of female (81.2 versus 66.7%, *P* < .001), hypertension (75.9 versus 62.5%, *P* < .001), less education (5.7 ± 4.3 vs. 8.0 ± 4.5, *P* < .001), and the prevalence of low BMD (71.8 versus 50.0%, *P* < .001) were higher in the abnormal MMSE group (MMSE score < 24) than in the normal MMSE group (MMSE score ≥ 24). In total, 361 (55.5%) subjects had reduced BMD; 197 (30.3%) had osteopenia and 164 (25.2%) had osteoporosis. The prevalence of osteoporosis was higher in women (19.8%) than in men (5.4%).

**Figure 1:**
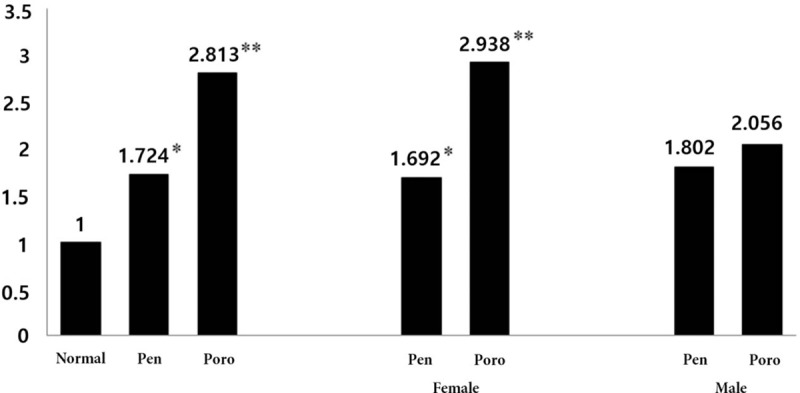
ORs for cognitive impairment, adjusted for age, sex, education, hypertension, diabetes and smoking, in the overall pool of subjects. Pen; osteopenia; Poro, osteoporosis; ^∗^*P* < .005; ^∗∗^*P* < .001. OR = odds ratio.

Table 2 and Figure 1 show the association of BMD and cognition in men and women. An unadjusted analysis showed that the odds ratio (OR) for cognitive impairment was 1.92 for subjects with osteopenia (95% confidence interval [CI] 1.24–2.99, *P* = .003) and 3.43 for those with osteoporosis (95% CI 2.21–5.33, *P* < .001). After adjustment for age, sex, education, and other confounding factors such as hypertension, diabetes mellitus, and smoking, the OR was 1.72 for osteopenia (95% CI 1.09–2.14, *P* = .019) and 2.81 for osteoporosis (95% CI 1.78–4.45, *P* < .001).

**Table 2:**
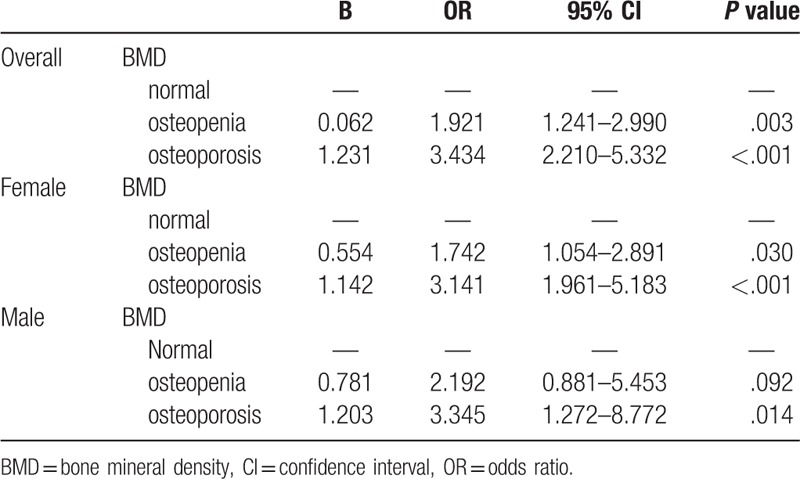
Results of univariate analysis for association between BMD and cognition.

The unadjusted analysis showed that the OR for cognitive impairment in women was 1.74 for subjects with osteopenia (95% 1.05–2.89, *P* = .030) and 3.14 for those with osteoporosis (95% CI 1.90–5.18, *P* < .001). After adjustment for age, sex, education, and other confounding factors such as hypertension, diabetes mellitus, and smoking, the OR was 1.69 for osteopenia (95% CI 1.00–2.84, *P* = .047) and 2.93 for osteoporosis (95% CI 1.73–4.92, *P* < .001). In men, the unadjusted OR for cognitive impairment was 2.19 for subjects with osteopenia (95% CI 0.88–5.45, *P* = .092) and 3.34 for those with osteoporosis (95% CI 1.27–8.77, *P* = .014). After adjustment for age, sex, education, and other confounding factors such as hypertension, diabetes mellitus, and smoking, the OR was 1.80 for osteopenia (95% CI 0.68–4.78, *P* = .230) and 2.05 for osteoporosis (95% CI 0.72–5.84, *P* = .176).

## 4 Discussion

Our results showed a significant association between cognitive impairment and low BMD in adults aged 50 years and above with no previous history of dementia or stroke. Furthermore, BMD showed an independent correlation with cognitive impairment, especially in women, after adjusting for confounding factors. This finding suggests that maintaining normal bone density might help in preventing cognitive impairment.

Possible explanations for the association of BMD with cognitive impairment include the following:

1. Estrogen, an important hormone in bone homeostasis, may affect cognitive function by various mechanisms.^[3,10–12]^ Estradiol, the most common type of estrogen measured in nonpregnant women, may decrease oxidative stress, inhibit neuronal apoptosis, and promote synaptogenesis and synaptic plasticity. Estrogen improves cerebral blood flow by increasing high-density lipoprotein cholesterol levels, thereby reducing atherosclerosis and inhibiting endothelin-mediated vasoconstriction. This is probably why estrogen replacement benefits cognitive function, especially in postmenopausal women.^[4,6]^
2. Abnormal electrolytes in osteoporosis may induce an influx of calcium and neuronal cell death, which facilitate the formation of senile plaques and neurofibrillary tangle in AD.^[3,5,13]^
3. Bone mass loss may increase inflammatory markers such as interleukin-6, which is an increased risk factor for AD.^[14,15]^ However, the relationship between BMD and dementia was mainly determined in women or the elderly with or without dementia, and there is a lack of consistency in this relationship among men.^[3–6]^ In our study, the relationship remained significant in women as in previous studies, but, in men, the relationship was inconsistent even after controlling for well-known vascular risk factors, although osteoporosis was associated with cognitive impairment in an unadjusted analysis (*P* = .014). This difference may be a result of slower bone loss and higher peak bone mass in men.^[14,16]^ Although this finding cannot be regarded as conclusive because of the small number of men in the study, the results are consistent with previous studies of sex differences in the association between BMD and cognition. Further research is needed to understand this phenomenon.

The purpose of our study was to evaluate bone mass loss as a controllable risk factor affecting cognitive function. However, our study has some limitations. First, this was a cohort-based cross-sectional, observational study. Since we could not determine the temporal relationship between low BMD and cognitive impairment, further research is needed on this question. Second, we assessed cognitive functions by using only the MMSE. The use of the MMSE alone is likely to produce inaccurate results for participants with low levels of education. Nevertheless, it is the most extensively used and characterized means for examining cognitive function. Third, there are other variables affecting BMD and cognitive function (e.g., exercise and menopause). However, we could not confirm the effects of these other confounding variables, because it was not possible to retrieve the medical history of all patients. Finally, there could have been a selection bias in the study subjects recruited by systematic random sampling in 2009, and in the inclusion of volunteers who were more likely to be of generally low socioeconomic status in 2010. Although there were no significant differences in demographic characteristics between these 2 groups, the study population may not have been representative of the general population. In addition, our study included more female than male participants, due to the fact that the proportion of women is higher than that of men among the elderly population, combined with the cultural tendency of men to participate less in research. Therefore, caution is needed when applying these findings to the general population.

## 5 Conclusion

In conclusion, we found that low BMD is correlated with cognitive impairment in community-dwelling, stroke-free, dementia-free adults, especially in women. On the basis of these findings, we believe that a community-based, early life, active preventive education campaign for osteoporosis might decrease the incidence of dementia in the community-dwelling elderly. However, this study should be considered as a preliminary study. Therefore, it is necessary to confirm the results of our study through a more systematic and larger-scaled prospective study.

## Author contributions

**Conceptualization:** Hyun Goo Kang, Hyun Young Park, Seung-Han Suk.

**Data curation:** Hyun Goo Kang, Hyun Young Park, Han Uk Ryu, Seung-Han Suk.

**Formal analysis:** Hyun Goo Kang, Hyun Young Park, Seung-Han Suk.

**Investigation:** Hyun Goo Kang, Han Uk Ryu, Seung-Han Suk.

**Methodology:** Hyun Goo Kang, Han Uk Ryu, Seung-Han Suk.

**Resources:** Hyun Goo Kang, Hyun Young Park, Seung-Han Suk.

**Validation:** Hyun Goo Kang, Hyun Young Park.

**Writing – original draft:** Hyun Goo Kang, Hyun Young Park.

**Writing – review & editing:** Hyun Goo Kang.

**Funding acquisition:** Hyun Young Park.

**Supervision:** Han Uk Ryu, Seung-Han Suk.

Seung-Han Suk orcid: 0000-0002-8732-5021.
